# Characteristics and outcome of a first acute myocardial infarction in patients with ankylosing spondylitis

**DOI:** 10.1007/s10067-020-05354-3

**Published:** 2020-08-26

**Authors:** Anna Södergren, Johan Askling, Karin Bengtsson, Helena Forsblad-d’Elia, Tomas Jernberg, Ulf Lindström, Lotta Ljung, Ängla Mantel, Lennart T. H. Jacobsson

**Affiliations:** 1 Department of Public Health and Clinical Medicine/Rheumatology, Umeå University, Umeå, Sweden; 2 Wallenberg Centre for Molecular Medicine (WCMM), Umeå University, Umeå, Sweden; 3 Clinical Epidemiology Section, Department of Medicine Solna, Karolinska Institutet, Stockholm, Sweden; 4 Department of Rheumatology and Inflammation Research, Sahlgrenska Academy at University of Gothenburg, Gothenburg, Sweden; 5 Department of Clinical Sciences, Danderyd University Hospital, Karolinska Institutet, Stockholm, Sweden

**Keywords:** Acute myocardial infarction, Ankylosing spondylitis, Cardiovascular disease, Mortality

## Abstract

**Objectives:**

To study clinical characteristics, mortality, and secondary prevention, after a first incident acute myocardial infarction (AMI) in patients with ankylosing spondylitis (AS) compared with the general population.

**Methods:**

In total, 292 subjects with AS and a first AMI between Jan 2006 and Dec 2014 were identified using the Swedish national patient register. Each subject was matched with up to 5 general population comparators per AS-patient (*n* = 1276). Follow-up started at the date of admission for AMI and extended until death or 365 days of follow-up. Cox regression was used to assess mortality in two time intervals: days 0–30 and days 31–365. For a subgroup with available data, clinical presentation at admission, course, treatment for AMI, and secondary prevention were compared.

**Results:**

During the 365-day follow-up, 56/292 (19%) AS patients and 184/1276 (14%) comparators died. There were no difference in mortality due to cardiovascular-related causes, although the overall mortality day 31–365 was increased among patients with AS compared with comparators (HR [95% CI] = 2.0 [1.3;3.0]). At admission, AS patients had a higher prevalence of cardiovascular comorbidities compared with comparators. At discharge, patients with AS were less often prescribed lipid-lowering drugs and non-aspirin antiplatelet therapy**.**

**Conclusions:**

Patients with AS tend to have a higher comorbidity burden at admission for first AMI. The mortality after a first AMI due to cardiovascular-related causes does not seem to be elevated, despite an increased overall mortality during days 31–365 among patients with AS compared with the general population.

**Table:** 

**Key Points** *• The all-cause mortality after a first AMI was higher in patients with AS.* *• Mortality after a first AMI due to CVD-related causes does not seem to be elevated for patients with AS.* *• In patients with AS suffering a first AMI, more attention should be given to other comorbidities causing an excess in mortality.*

**Electronic supplementary material:**

The online version of this article (10.1007/s10067-020-05354-3) contains supplementary material, which is available to authorized users.

## Introduction

Ankylosing spondylitis (AS) is a chronic, inflammatory disease, primarily involving the spine as well as peripheral joints and entheses [1]. Well-known cardiovascular complications of AS include cardiac conduction disorders and aortic regurgitation [2]. Recent studies have shown that patients with AS also have an increased risk of atherosclerotic comorbidity such as acute myocardial infarction (AMI) and stroke compared with the general population [3–5]. In total, patients with AS have a higher both overall and cardiovascular disease (CVD)–related mortality compared with the general population [6, 7].

Inflammation may have an impact on the development of accelerated atherosclerosis in AS [8], but an increased frequency of traditional risk factors (diabetes mellitus, hypertension, hyperlipidemia, and smoking) may also add to the risk of AMI [3, 9]. Moreover, there are indications that patients with chronic inflammatory diseases might have an altered outcome after an CVD event [3, 10, 11]. Since AS also affects the entheses of the thorax, chest pain is common among these patients as part of the rheumatic disease, which could result in an atypical clinical presentation that may be misinterpreted by both patients and caregivers [12]. The course and pattern of AMI in AS patients compared with general population controls have not previously been studied systematically.

With this background we wanted to study (1) clinical characteristics of as well as (2) CVD-related and general mortality after a first incident AMI and (3) the initiation and prescription of cardioprotective secondary preventive pharmacotherapies after first incident AMI in a population-based cohort of patients with prevalent AS compared with the general population.

## Method

### Design and setting

We performed a nationwide population-based cohort study of patients with prevalent AS experiencing a first ever AMI event, compared with a matched general population cohort. The study was based on prospectively recorded register data.

The decentralized and publicly funded Swedish healthcare system enables equal access to all healthcare services for all residents, including specialized care. A unique personal identification number (PIN) is assigned to all Swedish residents at birth or immigration, and can be used to link information from official registries and other data sources. With the PIN as key, several nationwide registers were linked to the identified cohorts.

### Data sources

The National Patient Register (NPR) was used to identify individuals with AS, AMI events, and pre-existing comorbidities of interest. NPR contains information on in-hospital care since 1964 (with full national coverage since 1987), as well as out-patient specialized care since 2001. NPR holds administrative information on specialized outpatient care and inpatient care, together with diagnoses coded according to the Swedish version of International Classification of Disease (ICD).

The cause-of-death register was used to retrieve information on mortality, such as dates and cause of death according to the ICD. The Population Register, provided by Statistics Sweden, was used to retrieve information, such as sex, age, civil status, and dates of migration.

The prescribed drug register (PDR) includes information on all dispensed drugs from Swedish pharmacies since 2005, coded by the Anatomical Therapeutic Chemicals (ATC) classification. PDR were used to define dispensed medications for AS (see Supplementary Table 1 for details).

**Fig. 1:**
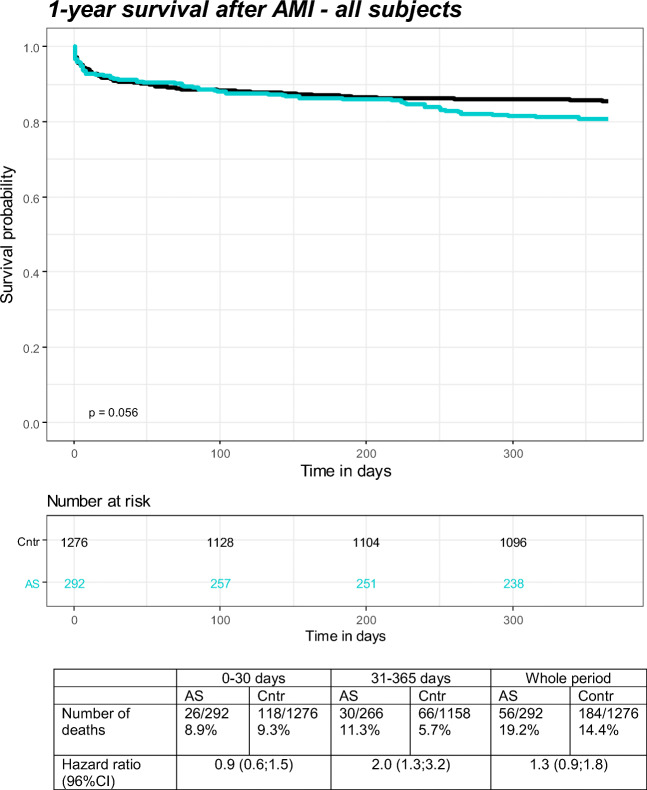
Kaplan-Meier survival curves, number of deaths, and HR (95% CI) after first AMI in patients with AS and general population comparators during 365-day follow-up. Data are presented for the whole period and for intervals; 0–30 and 31–365 days

The Swedish Web-system for Enhancement and Development of Evidence-based care in Heart disease Evaluated According to Recommended Therapies (SWEDEHEART) is a national quality of care register including patients admitted with AMI since 1995. This register was used to retrieve information on clinical presentation at admission, in-hospital treatments, complications, and status at discharge. SWEDEHEART includes information on risk factors, baseline characteristics, symptoms, in-hospital examinations, treatments, interventions, complications, discharge status, after-care, and secondary prevention (full protocol available at: http://www.ucr.uu.se/swedeheart/). Since 2005, SWEDEHEART also gather data on secondary prevention up to 1-year follow-up after the AMI. Today, SWEDEHEART has a coverage of more than 90%, but this has been increasing over the years. Approximately 85% (81% of patients with AS vs. 86% of general population comparators) of our study subjects who were diagnosed with AMI according to the NPR were also recorded and included in SWEDEHEART.

### Study populations

Subjects with AS were defined as follows: (1) ≥ 18 years of age (no upper age limit), (2) having a hospitalisation and/or out-patient visit with a registered diagnosis of AS (ICD-10 M45) in the NPR between Jan 2001 and Dec 2014, (3) not having any recorded visit in the NPR with an ICD-code for psoriatic arthritis (ICD-10 L405, M07.0–3), rheumatoid arthritis (ICD-10 M05, M06.0–9, or M123), systemic lupus erythematosus (ICD-10 M32.0–1 or M32.8–9), juvenile arthritis (ICD-10 M08 or M09), or reactive arthritis (M013–M029 or M036) during these years, in order to minimize risk of misclassification.

A first incident AMI (ICD-10 I21) after diagnosis of AS and from Jan 2006 through Dec 2014 was identified in the NPR in 292 of the patients with AS. For each of these 292 patients, up to five individually matched controls with a first incident AMI (matched by year of birth, sex, year of AMI, and area of residency by the year of the first incident AMI) were identified as comparators (*n* = 1276). The same inclusion and exclusion criteria were used as for all individuals (Supplementary Table 1). Only individuals admitted to a hospital for the AMI were detected. Individuals with sudden cardiac death were not included (Supplementary Table 1).

### Definitions of follow-up and outcomes

Pre-existing comorbidities (definitions and ICD-codes in Supplementary Table 1) were identified by a diagnosis in the NPR before the admission for the first AMI. Pre-existing pharmacotherapies were defined as a dispensed drug in the PDR 121–365 days prior to the AMI (ATC-codes in Supplementary Table 1).

The follow-up for both AS patients and general population comparators started at the date for admission for the first AMI event. All individuals were followed until death, emigration, or 365 days of follow-up after the AMI, whichever occurred first. Mortality (from any cause, including CVD) was defined as mortality within 30 days and days 31–365 after the AMI, and a secondary analysis was performed analysing mortality due to CVD (Supplementary Table 1).

All cases and matched comparators included in SWEDEHEART were compared regarding clinical presentation and treatment: symptoms at onset, blood pressure, heart failure (classified by Killip class), in-hospital treatment (coronary angiography and any primary reperfusion (thrombolysis, percutaneous coronary intervention, or coronary artery by-pass surgery)), ejection fraction (echocardiography), complications, and discharge diagnosis (ST-elevation myocardial infarction (STEMI) or non-ST-elevation myocardial infarction (non-STEMI) as registered by cardiologist based on ECG changes and laboratory data). Secondary preventive pharmacotherapies and smoking habits were assessed and registered in SWEDEHEART. For variables included in SWEDEHEART with different reference values during different time periods, for example biomarkers for myocardial damage, the most prevalent was chosen, i.e. TroponinT, which also was the most recent.

## Statistical analyses

Baseline data and variables used to characterize the AMI are presented as frequencies (percentage of individuals with available data, %) or means (standard deviation, SD) as appropriate. Differences between the groups were tested using chi^2^ tests, *t* test, or Mann-Whitney *U* test as appropriate. One-year time-to-event was summarized by Kaplan-Meier method and survival distributions were compared by log-rank test. We used Cox regression models, with time since AMI as timescale, adjusted for age and sex, to assess the hazard ratios (HRs) of mortality. The assumption of proportional hazards was assessed through inspection of survival curves and insertion of a time-interaction product in the models. Stratified analyses were performed by sex and STEMI/non-STEMI type of AMI event. A *p* value < 0.05 was considered statistically significant, and all *p* values were two sided. Missing data was regarded as random; however, where missing data were more than 25% that variable was excluded from further analysis, no imputation was made. Number of individuals with valid data on each variable is given in the “Results” section.

All analyses were carried out with SAS software package version 9.3 (SAS Institute, Cary, NC, USA) and the graphics were drawn in R 3.6.1. The study was conducted in accordance with the principles outlined in the Declaration of Helsinki and principles of Good Clinical Practice (GCP). The study protocol was approved by the Regional Ethics Committee in Stockholm, Sweden (registration number 2015/1844-31/2).

## Results

We identified 292 patients with AS and a first AMI, and 1276 matched general population comparators (Table 1). Patient characteristics and prevalence of comorbidities at admission for AMI are presented in Table 1. No statistically significant differences in body mass index (BMI) or smoking habits were observed for AS compared with population comparators, but most of the analysed comorbidities were more common in the patients with AS (Table 1). Patients with AS had also significantly more often been prescribed non-steroidal anti-inflammatory drugs (NSAIDs). Data on comorbidities for women and men, respectively, are given in Supplementary Tables 2a and 2b.

**Table 1:** Patient characteristics and occurrence of prevalent comorbidities in patients with AS and a first AMI and matched general population comparators at admission for the first AMI

	AS (*n* = 292)	General population comparators (*n* = 1276)	*p* value
Patient characteristics:			
Women, *n* (%)	46 (16%)	173 (14%)	0.33
Age, years	67.9 ± 11.1	68.1 ± 10.7	0.47
Smoking ever, *n* (%)	151/236 (64%)	646/1089 (59%)	0.18
BMI, kg/m^2^	27.4 ± 5.1 (*n* = 188)	27.4 ± 4.5 (*n* = 907)	0.81
AS-related extra-articular disease manifestations before AMI:			
Anterior uveitis, *n* (%)	65/292 (22)	8/1276 (1)	< 0.0001
Inflammatory bowel disease, *n* (%)	34/292 (12)	16/1276 (1)	< 0.0001
Cardiovascular comorbidities before AMI:			
Stable ischemic heart disease, n (%)	84/292 (29)	229/1276 (18)	< 0.0001
Congestive heart failure, *n* (%)	38/292 (13)	81/1276 (6)	0.0001
Cerebrovascular events, *n* (%)	43/292 (15)	137/1276 (11)	0.05
Cardiac valve disease, *n* (%)	22/292 (8)	34/1276 (3)	< 0.0001
Atrial fibrillation, *n* (%)	38/292 (13)	129/1276 (10)	0.15
Other comorbidities before AMI:			
Pulmonary diseases, *n* (%)	25/292 (9)	70/1276 (5.5)	0.047
Thromboembolic disease, *n* (%)	13/292 (5)	25/1276 (2)	0.01
Diabetes mellitus, *n* (%)	55/292 (19)	172/1276 (14)	0.02
Hypertension, *n* (%)	157/292 (54)	402/1276 (32)	< 0.0001
Malignancy, *n* (%)	60/292 (21)	218/1276 (17)	0.16
Renal disease, *n* (%)	55/292 (19)	120/1276 (9)	< 0.0001
Hospitalisation during the last 2 years, days	11.6 ± 27.0 (*n* = 292)	5.0 ± 19.3 (*n* = 1276)	< 0.0001
Treatment related to AS 121–365 days prior to the AMI:			
NSAID, *n* (%)	80/292 (27)	101/1276 (8)	< 0.0001
csDMARD, *n* (%)	29/292 (10)	7/1276 (1)	< 0.0001
bDMARD, *n* (%)	16/292 (6)	1 (0.1)	< 0.0001

Data are presented as *n* (%) or mean value ± SD and the differences in frequencies in comorbidities and disease manifestations were assessed using chi-square test

*AS* ankylosing spondylitis, *SD* standard deviation, *NSAID* non-steroidal anti-inflammatory drugs, *csDMARD* conventional synthetic disease-modifying antirheumatic drug (e.g. methotrexate, sulfasalazine, leflunomide), *bDMARD* biologic disease-modifying antirheumatic drug (i.e. etanercept, adalimumab, infliximab)

For individuals from the cohorts that were also identified in SWEDEHEART (*n* = 237 (81%), *n* women = 34 (14%), among the patients with AS; *n* = 1093 (86%), *n* women = 122 (11%) among the matched general population comparators), the mean age was 66.7 ± 10.6 years for AS and 67.1 ± 10.1 years for general population comparators.

### Characteristics of the first AMI

There were no statistically significant differences in infarction classification according to non-STEMI and STEMI respectively, in patients with AS compared with general population comparators (Table 2). Moreover, the characteristics of the first AMI, including time to admission and onset symptoms, were similar between groups (Table 2).

**Table 2:** Characteristics of, and care given after, at first AMI in patients with AS and matched general population comparators based on the subgroups recorded in SWEDEHEART

	AS (n = 237)	General population comparators (*n* = 1093)	*p* value
Clinical presentation:			
Symptom until ECG, h	7.4 ± 37.7	10.6 ± 77.5	0.70
Symptom onset: dyspnoea, *n* (%)	10/210 (4.8%)	60/1001 (6.0%)	0.49
Symptom onset: Cardiac arrest, *n* (%)	4/210 (1.9%)	16/1001 (1.6%)	0.75
Prehospital cardiopulmonary resuscitation, *n* (%)	4/223 (1.8%)	22/1048 (2.1%)	0.76
Chest pain at onset, *n* (%)	196/210 (93.3%)	925/1001 (92.4%)	0.64
ST-elevation myocardial infarction (STEMI), *n* (%)	53/175 (30.3%)	301/827 (36.4%)	0.12
Non-ST-elevation myocardial infarction (non-STEMI), *n* (%)	99/175 (56.6%)	436/827 (52.7%)	0.35
Atrial fibrillation, *n* (%)	26/236 (11.0%)	90/1089 (8.3%)	0.18
High sensitive TroponinT, maximal value (ng/L)	2191 ± 3880	1753 ± 2651	0.24
Killip class			0.09
1	116/123 (96.7%)	545/585 (93.2%)	
2	2/123 (1.7%)	25/585 (4.3%)	
3	0	6/585 (1.0%)	
4	2/123 (1.7%)	6/585 (1.0%)	
In-hospital course:			
Duration of hospitalisation, days	5.3 ± 5.3	5.3 ± 5.8	0.99
Any primary reperfusion in individuals with STEMI, *n* (%)	49/53 (92.5%)	256/301 (85.3%)	0.16
Cardiogenic shock, *n* (%)	4/230 (1.4%)	14/1064 (1.3%)	0.62
Examination:			
Three vessel disease on coronary angiography, *n* (%)	56/224 (24.9%)	231/1044 (22.0%)	0.37
Low ejection fraction, < 40%, *n* (%)	24/160 (15.0%)	158/821 (19.2%)	0.21

Results are presented as *n* (%) or mean value ± SD. *p* values are calculated by chi-square test comparing AS and general population comparators

Primary reperfusion includes PCI, CABG, and thrombolysis

The Killip classification is a risk assessment based upon clinical findings of heart failure in patients presenting with AMI (1 no signs of heart failure; 2 third heart sound, elevated jugular pulse, rales in < 50% of posterior lung fields; 3 overt pulmonary oedema; 4 cardiogenic shock)

## Mortality

The patients with AS were followed for 251.4 person years, whereas the general population comparators were followed for 1118.3 person years giving 22.3 (17.1–29.0) deaths/100 person years among patients with AS and 16.5 (14.2–19.0) deaths/100 person years among general population comparators (*p* = 0.05). During the 365 days of follow-up, 19.2% (56/292) of the patients with AS and 14.4% (184/1276) of the general population comparators died (Fig. 1). Over the first 30 days, the corresponding mortality was 8.9% (26/292 patients with AS) and 9.3% (118/1276 comparators). The 1-year mortality after AMI among women with AS was 23.9% (11/46) compared with 22.5% (39/173) in the general population, and among men 18.3% (45/246 patients with AS) and 13.2% (145/1103 comparators).

Patients with AS did not have a statistically significant increased mortality during the first 30 days after AMI (HR [95% CI] = 0.9 [0.6;1.5]) nor during the whole first year (HR [95% CI] = 1.3 [0.9;1.8]). However, over the 31–365-day period, the mortality was significantly increased (HR [95% CI] = 2.0 [1.3;3.2]).

The major reported cause of death among both patients with AS and general population comparators (55% vs. 74%, *p* < 0.05) was CVD-related (sudden cardiac arrest, arrhythmias, heart failure, or other complications related to CVD). The CVD-related mortality during days 0–30 was comparable among patients with AS (20/292, 6.8%) and the general population (101/1276, 7.9%) (HR (95% CI) = 1.2 [0.7;1.9]). Also, during days 31–365, CVD-related mortality was comparable among patients with AS (11/292, 3.8%) and the general population (36/1276, 2.8%) (HR (95% CI) = 0.99 [0.66; 1.5]).

The assumption of proportional hazards was fulfilled for the first 30 days but was violated for the whole 1-year follow-up as well as the day 31–365 follow-up. Time-stratified analysis indicated proportional hazards up to approximately day 220, with no difference in hazard rate during days 0–220.

Stratified analysis by sex and STEMI/ non-STEMI, respectively, showed no differences in 1-year mortality after a first AMI between AS patients and the general population comparators in any of the subgroups (data not shown).

### Prescriptions of CVD-related pharmacological agents prior to admission and at discharge after first AMI

Prior to admission for the first AMI, patients with AS had been prescribed CVD medications at a similar or higher frequency as the general population comparators (Table 3). At discharge, patients with AS were prescribed lipid-lowering agents and/or non-aspirin antiplatelet therapy less often than general population comparators (Table 3). Slow releasing nitro-glycerin, diuretics, and calcium channel blockers were on the other hand prescribed more often in patients with AS compared with general population comparators both before and after the AMI (Table 3).

**Table 3:** Pharmacological treatment prior to and at discharge of a first AMI in patients with AS and matched general population comparators. Data are given for individuals included in SWEDEHEART only

	On-going at admission			Prescribed at discharge		
Variable, *n* (%)	Patients with AS (*n* = 237)	General population comparators (*n* = 1093)	*p* value	Patients with AS (*n* = 237)	General population comparators (*n* = 1093)	*p* value
Lipid-lowering drugs	56/235 (23.9%)	226/1087 (20.8%)	0.28	185/233 (79.4%)	957/1093 (86.8%)	0.004
Slow release nitro-glycerin	20/233 (8.6%)	47/1093 (4.3%)	0.007	38/235 (16.2%)	77/1093 (7.0%)	< 0.0001
Aldosterone inhibitors	2/77 (2.6%)	5/417 (1.2%)	0.36	6/81 (7.4%)	24/407 (5.9%)	0.60
Diuretics	49/233 (21.0%)	167/1085 (15.4%)	0.03	66/235 (28.2%)	221/1049 (20.1%)	0.006
Digoxin	4/235 (1.7%)	11/1093 (1.0%)	0.36	4/235 (1.7%)	16/1067 (1.5%)	0.80
Calcium channel blockers	56/236 (22.8%)	156/1085 (14.5%)	0.001	54/234 (23.1%)	159/1093 (14.5%)	0.001
Beta-blockers	75/232 (32.3%)	271/1088 (24.9%)	0.02	195/235 (83.0%)	940/1093 (85.2%)	0.38
Non-aspirin antiplatelet therapy	8/235 (3.4%)	36/1091 (3.3%)	0.93	148/235 (63.0%)	841/1104 (76.2%)	< 0.0001
Low-dose aspirin	63/234 (26.9%)	244/1089 (22.4%)	0.13	205/235 (87.2%)	992/1093 (89.9%)	0.24
ACE/AII inhibitors	71/233 (30.5%)	280/1089 (25.7%)	0.14	162/234 (69.2%)	787/1093 (71.6%)	0.48

Results are presented as *n* (%), and the *p* values are calculated by chi-square test comparing patients with AS and general population comparators at the same timepoints

### CVD risk factors 1 year after admission for first AMI

When analysing the subpopulation with follow-up data on CVD risk factors 1 year after admission, using the SWEDEHEART register for follow up 1 year after AMI (data available in 702/1568 (44.8%)), there were no statistically significant differences between the two groups in blood pressure, cholesterol, HDL, LDL, Apo B/ApoA1, p-glucose, and HbA1c. However, serum triglycerides were on average slightly higher among patients with AS (mean value ± SD 1.5 ± 0.8 in AS and 1.4 ± 0.8 in general population, *p* < 0.05).

## Discussion

In this population-based and nationwide cohort study, the 30-day all-cause mortality after AMI in patients with AS did not differ from that in general population. Also, when taking only CVD-related death into account, there was no difference in mortality between patients with AS and general population comparators. However, mortality day 31 through 365 was increased in patients with AS. Rather than an inferior CV outcome in AS, this difference in HR in short- and long-time follow-up after AMI may be regarded as a return to the baseline risk, based on the well-documented overall increased mortality risk in patients with AS [6, 7, 13, 14].

At admission, the clinical history in patients with AS differed in several aspects from that in general population, in particular with regard to an increased occurrence of CVD, but also other comorbidities such as pulmonary disease, renal disease, diabetes mellitus, and history of serious infections. Such increased prevalence of comorbidities at admission for AMI in rheumatic disease has been shown before [11, 15]. This increase could partly be explained by a detection bias due to an increased awareness and screening for comorbidities in the AS patients. On the other hand, it has also been shown that comorbidities in chronic diseases are often undertreated [16]. Taken together, this increased burden of comorbidities could in part explain the increased mortality during longer follow-up in the present study, in particular since cause-specific death of CVD, the well-known leading cause for death in AS [6, 7, 13, 14], was not significantly increased in the AS patients.

It may seem counter-intuitive that the mortality days 0–30 did not differ between AS patients and controls, considering the higher occurrence of comorbidities in AS patients. However, for all individuals, the risk for death after an AMI is highest in close proximity to the infarction which may diminish the relative effect of underlying comorbidities. Also, there were no differences in onset of symptoms, delay between symptom onset until first ECG was taken, or interventions given during hospitalisation between patients with AS and general population comparators. This is further reflected in the lack of significant differences in CVD risk factors measured for secondary prevention after 1 year of follow up.

To our knowledge, there are no other studies describing short-term outcome and characteristics of AMI in patients with AS. In RA, we have in the same setting described an impaired outcome after the AMI, even after taking AMI type and comorbidities into account [10]. In the present study, there were no difference in the evaluated characteristics at discharge in patients with AS compared with the general population comparators. When considering patients with RA compared with general population, tendencies for a decreased myocardial function have been observed in RA [10]. AS has been associated with cardiac conduction disturbances and aortic regurgitation [2] and studies have also found an increased risk of atrial fibrillation in AS compared with general population [17, 18]. In the present analyses, with limited number of cases included on the basis of AMI, we could not detect any increase in atrial fibrillation although we, as expected, found an increased occurrence of cardiac valve disease at admission in AS patients. We also found an increased occurrence of diagnosed ischemic heart disease and congestive heart failure at admission in AS patients compared with the general population comparators, suggesting that this population may have a relatively poor long-term CVD outcome [19].

Treatment with non-steroidal anti-inflammatory drugs (NSAIDs) has been associated, through several mechanisms, with increased risk of adverse outcomes in patients with CVD [20]. In AS, treatment with NSAID is important for pain relief and to reduce musculoskeletal pain and stiffness. Among the patients with AS who experienced an AMI, 27% had been prescribed NSAIDS, which is considerably more than among the general population comparators. However, these numbers are slightly lower than the general prescription among patients with AS in the same setting, where the prescription of NSAIDs was 55.5% [21]. Both these estimates may underestimate the use of NSAID, since they only take into account dispensed prescriptions.

There are several limitations of the present study. First, due to differences in indication of admission to coronary intensive care units or other specialized facilities in patients over 80 years of age, the overall coverage of this age group in SWEDEHEART is lower. As a result, approximately one-fifth of the study population, similarly for AS and their comparators, were not included in the analysis of AMI treatment. Whereas this restriction will not in itself introduce bias, it may pose a limitation to the generalisability, especially in older patients. Second, the proportion that died from AMI or sudden cardiac death before being hospitalized is not known.

There are also several strengths. First, the use of nationwide patient registers with high reported validity and close to complete coverage enabled adequate identification of AS, AMI, relevant comorbidities, and other covariates [22]. We identified AMI, length of hospitalisation, and deaths from linkage to national and virtually complete register. Second, the accuracy of data entered in SWEDEHEART compared with data found in patient records is high [23], which argues against that misclassification of AMI would negatively influence internal validity. Third, the ICD code for AS in the NPR has previously been shown to have high positive predictive value for fulfilling both the modified New York criteria as well as the Assessment of SpondyloArthritis International Society (ASAS) classification criteria [24].

## Conclusions

The results of this study suggest that patients with AS have a higher comorbidity burden at admission for first AMI. Still, in this study, there was no increase in CVD-related mortality after a first AMI in patients with AS compared with general population, although the all-cause mortality was higher. There is a tendency for less use of cardioprotective drugs at discharge among the patients with AS. Taken together, this might indicate that in patients with AS suffering a first AMI, more attention should be given to other comorbidities causing an excess in mortality.

## Electronic supplementary material

ESM 1 (PDF 92 kb)

ESM 2 (PDF 74 kb)

## Data Availability

The data that support the findings of this study are available from Karolinska Institutet but restrictions apply to the availability of these data, which were used under licence for the current study, and so are not publicly available. Data are however available from the authors upon reasonable request and with permission of Karolinska Institutet.

## Abbreviations

AMI: acute myocardial infarction
AS: ankylosing spondylitis
ATC: Anatomical Therapeutic Chemicals Classification
bDMARD: biologic disease-modifying antirheumatic drug
BMI: body mass index
CABG: coronary artery by-pass surgery
CI: confidence interval
csDMARD: conventional synthetic disease-modifying antirheumatic drug
CVD: cardiovascular disease
ICD: International Classification of Disease
HR: hazard ratio
NPR: National Patient Register
NSAID: non-steroidal anti-inflammatory drugs
PCI: percutaneous coronary intervention
PDR: prescribed drug register
PIN: personal identification number
SD: standard deviation
STEMI: ST-elevation myocardial infarction
SWEDEHEART: Swedish Web-system for Enhancement and Development of Evidence-based care in Heart disease Evaluated According to Recommended Therapies
