# Supplementary material for: Characteristics and outcome of a first acute myocardial infarction in patients with ankylosing spondylitis
Source: Clin Rheumatol. 2020 Aug 26;40(4):1321–9. doi: 10.1007/s10067-020-05354-3 (PMC7943508; doi:10.1007/s10067-020-05354-3)
Supplement: Supplementary file 1 — (PDF 92 kb) [file 10067_2020_5354_MOESM1_ESM.pdf]

Characteristics and outcome of a first acute myocardial infarction in patients with ankylosing spondylitis

Clinical Rheumatology

Anna Södergren[1,5], Johan Askling[2], Karin Bengtsson[3], Helena Forsblad-d'Elia[1], Tomas Jernberg[4], Ulf Lindström[3], Lotta Ljung[1,2], Ängla Mantel [2], Lennart TH Jacobsson[3]

[1.] Department of Public Health and Clinical Medicine/Rheumatology, Umeå University, Umeå, Sweden, [2.] Clinical Epidemiology Section, Department of Medicine Solna, Karolinska Institutet, Stockholm, Sweden, [3.] Department of Rheumatology and Inflammation Research, Sahlgrenska Academy at University of Gothenburg, Gothenburg, Sweden, [4]. Dept of clinical sciences, Danderyd University Hospital, Karolinska Institutet, Stockholm, Sweden [5] Wallenberg Centre for Molecular Medicine (WCMM), Umeå University, Umeå, Sweden

Corresponding author: Anna Södergren, Dept. of Rheumatology, University Hospital, 901 85 Umeå, Sweden. Phone: +46 90 7851647; Email: anna.sodergren@umu.se

Supplementary table 1: Coding according to International Classification of Disease (ICD) or Anatomical Therapeutic Chemicals (ATC) for included, excluded diagnosis or pharmaceutical treatment respectively

**Included diagnoses**	Time period	ICD10
Ankylosing spondylitis (AS)	2001-2014	M45
Acute myocardial infarction (AMI)	2006-2014	I21

Excluded diagnoses patients with diagnoses 1968-2005 were excluded

**Category**	**from ICD10**	**to ICD10**	**from ICD9**	**to ICD9**	**from ICD8**	**to ICD8**
psoriatic arthritis	L405	L405	696A	696A	696,00	696,00
	M070	M071	713D	713D		
	M072	M072				
	M073	M073				
rheumatoid arthritis	M05	M059	714A	714C	712,10	712,10
	M060	M060	714W	714W	712,20	712,20
	M062	M063	719D	719D	712,38	712,39
	M068	M069				
	M123	M123				
reactive arthritis	M013	M029	711B	711B		
	M036	M036	711D	711W		
systemic lupus erythematosus	M320	M321	710A	710A	734,10	734,10
	M328	M329				
juvenile arthritis	M08	M09	714D	714D	712,0	712,0
myocardial infarction	I21	I22	410	410	410	410
unstable angina pectoris	I200	I200	411B	411B		

**AS-related co-morbidities before AMI:**	**from ICD10**	**to ICD10**	**from ICD9**	**to ICD9**	**from ICD8**	**to ICD8**
Anterior uveitis	H20	H20	364A	364B	364	364
	H221	H221				
Inflammatory bowel disease	K50	K51	555	556	563,00	563,00
					563,10	563,10
					569,02	569,02
**Cardiac co-morbidities before AMI:**						
Ischemic heart disease*	I20	I25	410	414	410	414
Congestive Heart Failure	I30	I52	420	429	420	429
Cerebrovascular events*	I60	I69	436	438	436	438
			430	434	430	434
			440	442	440	442

	G45	G45	433	435	435	435
Cardiac valve disease	I05	I08	394	397	394	396
	I34	I38	424A	424D	424,0	424,1
			424X	424X	424,9	424,9
	Q230	Q230	746D	746D	746,73	746,73
Atrial fibrillation	I48	I48	427D	427D	427,92	427,92
**Other co-morbidities before ACS:**						
Pulmonary diseases	J40	J47	490	496	490	490
					491,01	491,02
					491,04	491,04
					492	492
	J60	J64	500	505	491,01	491,02
	J66	J67	515	516	491,04	491,04
	J82	J82	518A	518D	492	493
	J84	J84			515	518
					519,20	519,22
Thromboembolic disease	I26	I26	415B	415B	450	453
			451	453		
	I80	I82	451	453	450	453
Diabetes mellitus	O24	O24	648A	648A		
	E10	E14	250	250	250	250
Hypertension	I10	I15	401	405	400	404
Infection, hospitalised	A00	B99	001	139	000	136
	G00	G02	320	322	320	320
	G042	G042	382	383	322	322
	G05	G07	460	466	381	383
	J00	J22	526E	526F	480	486
			480	487		
	N10	N10	590	590	590	590
Malignancy	C	C	140	239	140	239
Renal disease	N00	N19	580	594	580	594
**Causes of cardiovascular mortality**						
Ischemic heart disease	I20	I25				
Congestive Heart Failure	I30	I52				
Cerebrovascular events	I60	I69				
Peripheral artery disease	I71	I74				
Hypertension	E78	E78				

**Treatment related to AS before AMI:**	**ATC code**	**Generic name**
NSAID	M01A	
csDMARD		
	A07EC01	Sulfasalazine

	L04AD01	Cyclosporine
	L04AX01	Azathioprine
	L01BA01	Methotrexate
	L04AX03	Methotrexate
	M01CB01	Sodium aurothiomalate
	M01CB03	Auranofin
	P01BA01	Cloroquine
	P01BA02	Hydroxychloroquine
bDMARD		
	L01XC02	Rituximab
	L04AA24	Abatacept
	L04AB01	Etanercept
	L04AB02	Infliximab
	L04AB04	Adalimumab
	L04AB05	Certolizumabpegol
	L04AB06	Golimumab
	L04AC03	Anakinra
	L04AC07	Tocilizumab
