# Supplementary material for: Characteristics and outcome of a first acute myocardial infarction in patients with ankylosing spondylitis
Source: Clin Rheumatol. 2020 Aug 26;40(4):1321–9. doi: 10.1007/s10067-020-05354-3 (PMC7943508; doi:10.1007/s10067-020-05354-3)
Supplement: Supplementary file 2 — (PDF 74 kb) [file 10067_2020_5354_MOESM2_ESM.pdf]

Characteristics and outcome of a first acute myocardial infarction in patients with ankylosing spondylitis

Clinical Rheumatology

Anna Södergren[1,5], Johan Askling[2], Karin Bengtsson[3], Helena Forsblad-d'Elia[1], Tomas Jernberg[4], Ulf Lindström[3], Lotta Ljung[1,2], Ängla Mantel [2], Lennart TH Jacobsson[3]

[1.] Department of Public Health and Clinical Medicine/Rheumatology, Umeå University, Umeå, Sweden, [2.] Clinical Epidemiology Section, Department of Medicine Solna, Karolinska Institutet, Stockholm, Sweden, [3.] Department of Rheumatology and Inflammation Research, Sahlgrenska Academy at University of Gothenburg, Gothenburg, Sweden, [4]. Dept of clinical sciences, Danderyd University Hospital, Karolinska Institutet, Stockholm, Sweden [5] Wallenberg Centre for Molecular Medicine (WCMM), Umeå University, Umeå, Sweden

Corresponding author: Anna Södergren, Dept. of Rheumatology, University Hospital, 901 85 Umeå, Sweden. Phone: +46 90 7851647; Email: anna.sodergren@umu.se

Supplementary table 2a: Occurrence of prevalent co-morbidities in women with AS and a first AMI and matched general population comparators at admission for the first AMI. Data are presented as n (%) and the differences in frequencies in co-morbidities and disease manifestations were assessed using chi-square test.

	**AS (n=46)**		**Controls (n=173)**		**p-value**
	**N**	**%**	**N**	**%**	
Anterior uveitis	4	8.7	0	0	<.0001
Inflammatory bowel disease	2	4.4	3	1.7	0.3
Ischemic heart disease	14	30.4	30	17.3	0.05
Congestive Heart Failure	7	12.2	14	8.1	0.1
Cerebrovascular events	11	23	24	14	0.1
Cardiac valve disease	1	2.2	8	4.6	0.5
Atrial fibrillation	7	15.2	23	13.3	0.7
Arrhythmia	2	4.4	5	2.9	0.6
Pulmonary diseases	6	13.0	14	8.1	0.3
Thrombotic disease	0	0	4	2.3	0.3
Diabetes mellitus	7	15.2	33	19.1	0.5
Hypertension	27	58.7	69	39.9	0.02
Malignancy	14	30.4	79	28.3	0.8
Renal disease	7	15.2	12	6.9	0.08

Supplementary table 2b: Occurrence of prevalent co-morbidities in men with AS and a first AMI and matched general population comparators at admission for the first AMI. Data are presented as n (%) and the differences in frequencies in co-morbidities and disease manifestations were assessed using chi-square test.

	**AS (n=246)**		**Controls (n=1103)**		**p-value**
	**N**	**%**	**N**	**%**	
Anterior uveitis	61	24.8	8	0.7	<.0001
Inflammatory bowel disease	32	13.0	13	1.2	<.0001
Ischemic heart disease	70	28.5	199	18.0	0.0002
Congestive Heart Failure	31	12.6	67	6.1	0.0004
Cerebrovascular events	32	13.0	113	10.2	0.2
Cardiac valve disease	21	8.5	26	2.4	<.0001
Atrial fibrillation	31	12.6	106	9.6	0.2
Arrhythmia	7	2.9	20	1.8	0.3
Pulmonary diseases	19	7.7	56	5.1	0.1
Thrombotic disease	13	5.3	21	1.9	0.002
Diabetes mellitus	48	19.5	139	12.6	0.005
Hypertension	130	52.9	333	30.2	<.0001
Malignancy	46	18.7	169	15.3	0.2
Renal disease	48	19.5	108	9.8	<.0001
